# ZmRAD51C Is Essential for Double-Strand Break Repair and Homologous Recombination in Maize Meiosis

**DOI:** 10.3390/ijms20215513

**Published:** 2019-11-05

**Authors:** Juli Jing, Ting Zhang, Yazhong Wang, Zhenhai Cui, Yan He

**Affiliations:** 1 Joint Laboratory for International Cooperation in Crop Molecular Breeding, Ministry of Education, National Maize Improvement Center, China Agricultural University, Beijing 100094, China; jjlcurie@cau.edu.cn (J.J.); zhangting@cau.edu.cn (T.Z.); wangyazhong917@gmail.com (Y.W.); 2 College of Biological Science and Technology, Liaoning Province Research Center of Plant Genetic Engineering Technology, Shenyang Key Laboratory of Maize Genomic Selection Breeding, Shenyang Agricultural University, Shenyang 110866, China

**Keywords:** maize, meiosis, homologous recombination, double-strand break (DSB) repair, RAD51C

## Abstract

*Radiation sensitive 51* (RAD51) recombinases play crucial roles in meiotic double-strand break (DSB) repair mediated by homologous recombination (HR) to ensure the correct segregation of homologous chromosomes. In this study, we identified the meiotic functions of *ZmRAD51C*, the maize homolog of *Arabidopsis* and rice *RAD51C*. The *Zmrad51c* mutants exhibited regular vegetative growth but complete sterility for both male and female inflorescence. However, the mutants showed hypersensitivity to DNA damage by mitomycin C. Cytological analysis indicated that homologous chromosome pairing and synapsis were rigorously inhibited, and meiotic chromosomes were often entangled from diplotene to metaphase I, leading to chromosome fragmentation at anaphase I. Immunofluorescence analysis showed that although the signals of the axial element *absence of first division* (AFD1) and *asynaptic1* (ASY1) were normal, the assembly of the central element *zipper1* (ZYP1) was severely disrupted. The DSB formation was normal in *Zmrad51c* meiocytes, symbolized by the regular occurrence of γH2AX signals. However, RAD51 and *disrupted meiotic cDNA 1* (DMC1) signals were never detected at the early stage of prophase I in the mutant. Taken together, our results indicate that *ZmRAD51C* functions crucially for both meiotic DSB repair and homologous recombination in maize.

## 1. Introduction

In sexually reproducing eukaryotes, meiosis is a two-step cell division process with one round of DNA replication followed with two consecutive rounds of cell division to ensure the genetic exchange of parents and the formation of haploid generative cells [1,2]. During meiosis I, the physical connections between homologous chromosomes guarantee the proper reductional segregation and create new combinations of alleles [1,3,4].

Meiotic homologous recombination (HR) is initiated by programmed induction of DSB, which is catalyzed by an evolutionarily conserved Spo11 protein assisted with several accessary proteins [2,3,5]. To ensure proper HR repair, the MRX protein complex (Mre11–Rad50–Nbs1/Xrs2) combined with Sae2(Com1)/CtIP/Ctp1 resects dsDNA ends to release Spo11 oligonucleotides and creates 3′ single-stranded DNA tails [6,7,8,9,10,11,12,13]. Then the RecA recombinases RAD51 and DMC1 bind to the single-strand DNA tails, forming nucleo-protein filaments to promote the homology search and single strand invasion [14,15,16]. Ultimately, the resolution of strand-exchange intermediates generates either crossovers (COs) or non-crossovers products [17,18]. 

The eukaryotic Rad51 is homologous to the bacterial RecA and archaeal and yeast Rad51 [15,19,20]. With its DNA-dependent ATPase activity, Rad51 catalyzes the DNA strand exchange and is involved in meiotic and mitotic recombination [15]. Rad51 has a widely conserved function from yeast to humans [21]. Sharing about 20% to 30% amino acid sequence similarity with Rad51, seven Rad51-like proteins have been identified in vertebrates, namely, RAD51, RAD51B, RAD51C, RAD51D, DMC1, XRCC2, and XRCC3 [21,22,23,24,25,26,27]. Through co-immunoprecipitation and yeast, two hybrid studies, physical interactions between RAD51 paralogs are detected as two major complexes: RAD51C-XRCC3 (CX3) and RAD51B-RAD51C-RAD51D-XRCC2 (BCDX2) [28,29,30]. RAD51C is the only paralog detected in both two complexes, suggesting the more prominent role of RAD51C in homologous recombination [31]. Further biochemical studies have revealed the accurate function of human and mammalian RAD51C in DSB repair and recombination [32,33,34,35]. Similar to RAD51, RAD51C also exhibits DNA-stimulated ATPase activity that helps it bind to ssDNA and promote single strand invasion [35]. Then, RAD51C in complex with XRCC3 stabilizes RAD51-ssDNA nucleofilaments [29,36,37,38]. On the other hand, it was demonstrated that RAD51C is involved in double Holliday junctions (dHJ), branch migration and resolution in mammalian cells, which is important for HR intermediate processing [32]. 

Seven RAD51 homologs have been found in plants, and can be divided into two ancient clades, the *RADα* and *RADβ* subfamilies [39]. The *RADα* subfamily contains RAD51 and DMC1, whereas the RADβ subfamily consists of RAD51B, RAD51C, RAD51D, XRCC2, and XRCC3, which are also designated as the five RAD51 paralogs. In Arabidopsis, the loss of function of these RAD51 paralogues caused severe abnormalities in meiosis, such as chromosome fragmentation and defective homologue pairing and synapsis [31,40,41,42,43]. Similarly, in rice, meiosis was disturbed in two mutants harboring the defective RAD51 paralogues, *OsRAD51C* and *XRCC3* [44,45,46]. 

Maize (*Zea mays*) is one of the most prevalent crops worldwide and has been used as a model organism for the cytogenetic study for nearly a century [47,48,49,50,51,52,53]. However, none of the five maize RAD51 paralogues have been characterized yet. In this study, the maize *RAD51C* gene was isolated using a map-based cloning method. Cytological analyses demonstrated that *ZmRAD51C* is crucially required for the processing and repairing of DSBs, homologous chromosome pairing, and synaptonemal complex (SC) assembly in maize meiosis.

## 2. Results

### 2.1. Characterization of a Sterile Mutant

**Figure 1:**
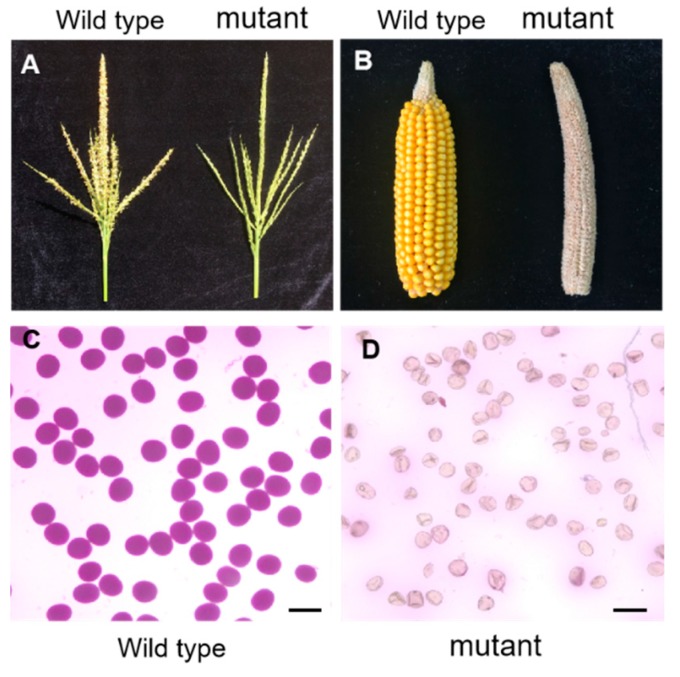
Phenotypic characterization of the wild type and mutant. (**A**) Comparison of a wild type tassel (left) and a mutant tassel (right); (**B**) Comparison of a wild type ear and a mutant ear after being pollinated with wild type male pollen grains; (**C**) Alexander-staining of normal pollen grains in the wild type; (**D**) Alexander-staining of sterile pollen grains in the mutant. Scale bar = 100 µm.

A sterile maize mutant was originally obtained from a mutant library created by ethylmethane sulfonate (EMS) mutagenesis in the Mo17 inbred line. The mutant was completely male-sterile in tassel (Figure 1A). When the mutant ear was pollinated with mature pollens from wild type plants, it failed to produce any seeds (Figure 1B), suggesting that the mutant was also female-sterile. To further examine male sterility, pollen grains from wild type and mutant plants were stained by Alexander solution, a common pollen viability stain [54]. Pollen grains from wild type showed the regular purple round shape (Figure 1C). In contrast, pollen grains from mutant plants were empty and shrunken, and failed to be stained (Figure 1D). Moreover, in the progeny of the self-pollinated heterozygous mutant plants, the segregation ratio of fertile (164) to sterile (53) plants fitted the 3:1 ratio (*χ^2^* =0.0384; *p* > 0.05), implying that the sterile phenotype of this mutant is caused by a single recessive mutation.

### 2.2. Map-Based Cloning and Characterization of ZmRAD51C

**Figure 2:**
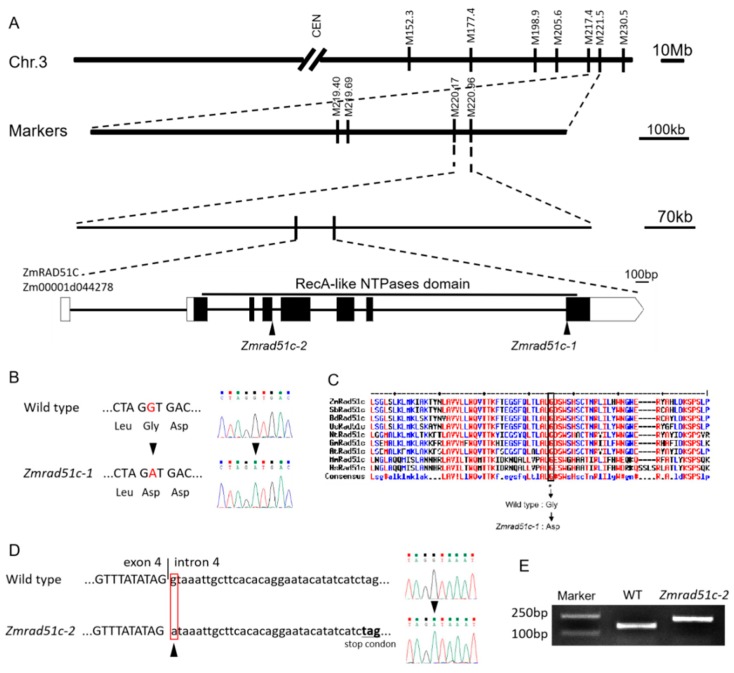
Map-based cloning of *ZmRAD51C*. (**A**) The procedure of map-based cloning of *ZmRAD51C*. CEN, centromere; M, markers. The mutation locus was mapped to a 787 kb region on the long arm of chromosome 3 using a total number of 457 segregated mutant plants of F_2_ generation. (**B**) Sequence analysis detected a single nucleotide substitution in the 8^th^ exon in the ORF of *ZmRAD51c* from G in wild type to A in *Zmrad51c-1*, leading to an amino acid substitution from Gly to Asp. (**C**) Multiple alignment of ZmRAD51C protein sequences for 9 different species. The substitution site of Gly to Asp is located on the conserved motif of the RecA-like NTPases domain. Species abbreviation: Zm, *Zea mays*; Sb, *Sorghum bicolor*, Bd, *Brachypodium distachyon*; Os, *Oryza sativa*; Nt, *Nicotiana tabacum*; Gm, *Glycine max*; At, *Arabidopsis thaliana*; Mm, *Mus musculus*; Hs, *Homo sapiens.* (**D**) Sequence analysis detected a single nucleotide substitution from G in wild type to A in *Zmrad51c-1* at the splicing donor site of the 4^th^ intron in the ORF of *ZmRAD51C*. (**E**) RT-PCR analysis demonstrated that the single nucleotide mutation in *Zmrad51c-2* allele caused the 4^th^ intron to be mis-spliced into the mature mRNA, producing a longer transcript.

A map-based cloning approach was used to isolate the mutated gene underlying this sterile mutant. An F_2_ mapping population was constructed by crossing the heterozygous mutant plants with the other inbred line B73. The candidate gene was primarily mapped to a 4.1 Mb region between M217.4 (Contig1365318) and M221.5 (Contig839180) on the long arm of chromosome 3 using 44 F_2_ mutant segregates showing the sterile phenotype (Figure 2A), then further delimited to a region of 787 kb by using the other 413 F_2_ mutant plants. Within this region, one candidate gene (*Zm00001d044278*) was found to harbor a G-to-A substitution at nucleotide position 629 in the coding sequence (CDS; Figure 2B), resulting in an amino acid substitution from Gly to Asp in the conserved RecA-like NTPases domain of a putative RAD51C homolog (Figure 2C). The phylogenetic tree further suggested that the predicted protein encoded by *Zm00001d046970* is the closest homolog of *RAD51C* in maize (Figure 2 and Figure S1). Therefore, we suspected this amino acid change on the conserved domain resulting from the mutation of *ZmRAD51C*, which finally led to maize sterility in the mutant. We named this mutant allele as *Zmrad51c-1.*

To further verify if the *Zmrad51c* mutation was responsible for the sterile phenotype, we obtained another mutant allele (EMS4-05639c) at the B73 inbred line background from the Maize EMS induced Mutant Database (MEMD) (http://www.elabcaas.cn/memd/) [55]. Sequencing revealed that this mutant allele contains a nucleotide substitution from G to A at the splicing donor site of the 4^th^ intron (Figure 2D). The RT-PCR analysis further confirmed that this single nucleotide mutation indeed resulted in the mis-splice of the 4^th^ intron into the mature mRNA (Figure 2E), and consequently produced a longer transcript with an in-frame premature stop codon (underlined tga) (Figure 2D). Therefore, this mutant allele was named as *Zmrad51c-2*. The homozygous plants of *Zmrad51c-2* also exhibited the same abnormal cytological behavior as *Zmrad51c-1* (Figure S2). Taken together, these results indicated that the sterility phenotype of the mutants was a result of the disruption of the *ZmRAD51C* gene.

Utilizing reverse transcription PCR, we obtained the full-length cDNA sequence of *ZmRAD51C*, which is composed of 8 exons and 7 introns and encodes a protein with 268 amino acids. According to the conserved domain search in NCBI (https://www.ncbi.nlm.nih.gov/Structure/cdd/wrpsb.cgi), ZmRAD51C protein contains a conserved RecA-like-NTPase domain, Walker A and B motif, multimer (BRC) interface, and ATP binding sites (Figure S3). The multiple alignment using the full-length amino acid sequence of RAD51C in 9 different species revealed that ZmRAD51C is evolutionarily conserved among different species (Figure S3). The spatio-temporal expression pattern of *ZmRAD51C* was analyzed by quantitative RT-PCR analyses. The results showed that *ZmRAD51C* was highly expressed in the developing anther, ear, embryo, and endosperm, but weakly expressed in root, stem, and leaf (Figure S4). 

### 2.3. Abnormal Meiotic Chromosome Behaviors in Zmrad51c 

**Figure 3:**
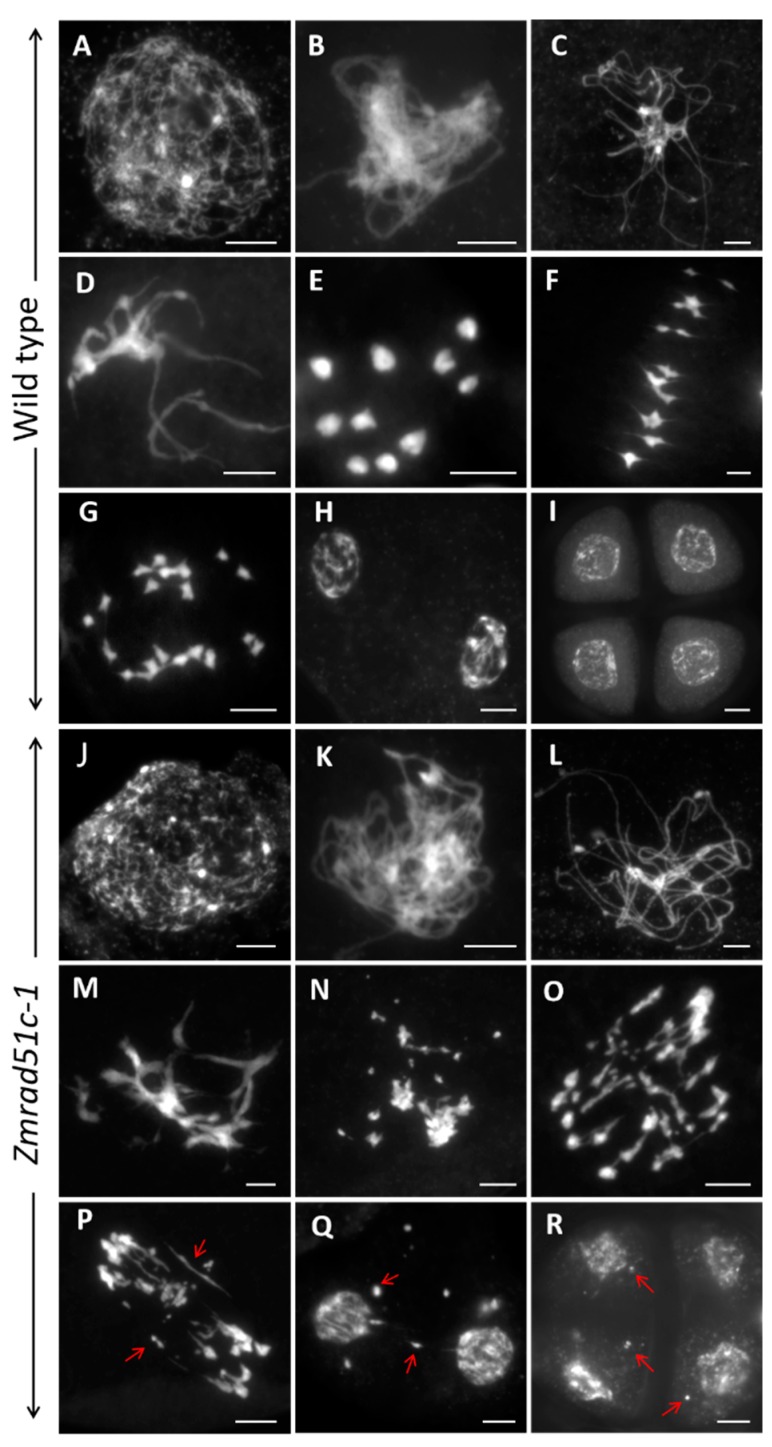
Male meiotic chromosome behaviors in wild type and *Zmrad51c-1* meiocytes. (**A**–**I**) Meiosis in wild type. (**A**) Leptotene; (**B**) zygotene; (**C**) pachytene; (**D**) late pachytene; (**E**) diakinesis; (**F**) metaphase I; (**G**) anaphase I; (**H**) telophase; (**I**) tetrads. (**J**–**R**) Meiosis in *Zmrad51c-1*. (**J**) leptotene; (**K**) zygotene; (**L**) pachytene; (**M**) late pachytene; (**N**) diakinesis; (**O**) metaphase I; (**P**) anaphase I; (**Q**) telophase; (**R**) tetrads. The red arrows indicated the abnormal chromosomal fragments, bridges, and micronuclei. Scale bars = 10 µm.

To clarify the reason for the sterility phenotype of *Zmrad51c* mutants, the meiotic chromosome behaviors in different stages of pollen mother cells (PMCs) were examined in both wild type and *Zmrad51c* mutants. In wild type meiocytes, the chromosomes began to condense as long thin threads at leptotene (Figure 3A). Then the homologous chromosomes came close to pairing and synapsis at zygotene (Figure 3B). During pachytene, homologs became tightly associated and synapsis was completed to form thick thread-like chromosomes (Figure 3C), which then gradually condensed in late pachytene (Figure 3D). During diakinesis, the paired chromosomes were highly condensed, forming 10 rod-shape or ring-shape bivalents (Figure 3E), which then aligned on the equatorial plate during metaphase I (Figure 3F). Subsequently, homologous chromosomes separated equally and migrated into two opposite poles of cells from anaphase I to telophase I (Figure 3G), producing a dyad with the same numbers of chromosomes (Figure 3H). During meiosis II, sister chromatids in the dyad separated and underwent cell division to produce tetrad microspores (Figure 3I).

In *Zmrad51c* meiocytes, chromosome behavior was indistinguishable from wild type in leptotene (Figure 3J and Figure S2A). However, chromosomes were observed as single threads without obvious pairing and synapsis from zygotene (Figure 3K and Figure S2B) to pachytene (Figure 3L and Figure S2C). At late pachytene, chromosomes gradually condensed but became an entangled mass throughout the nucleus (Figure 3M and Figure S2D), and bivalents were never observed (Figure 3N and Figure S2E) at diakinesis. At metaphase I, a few pieces of chromosome fragments were present and broken away from the main chromosome mass (Figure 3O and Figure S2F). During anaphase I, despite genome fragmentation, the entangled chromosome masses separated randomly, leading to asymmetric migration of the chromosomes to the opposite poles (Figure 3P and Figure S2G). Meanwhile, chromosome bridges were clearly visible between the separated chromosomes and chromosome fragments were randomly distributed throughout the nucleus (Figure 3P and Figure S2G. At telophase I, many chromosome fragments were lagged and scattered in the nucleus of the dyad (Figure 3Q and Figure S2H). After the second meiosis division, several chromosome fragments and micronuclei were always dispersed in tetrads (Figure 3R and Figure S2I). Therefore, we concluded that the abnormal chromosome synapsis and the appearance of chromosomal fragmentation are responsible for the complete sterility of *Zmrad51c* mutants. Since *Zmrad51c-1* and *Zmrad51c-2* exhibited the same defect in the meiotic chromosome behaviors, all subsequent analyses were conducted using *Zmrad51c -1* mutant as representative of the *Zmrad51c* defect. 

### 2.4. Normal Telomere Bouquet Clustering but Deficient Homologous Chromosome Pairing in Zmrad51c

**Figure 4:**
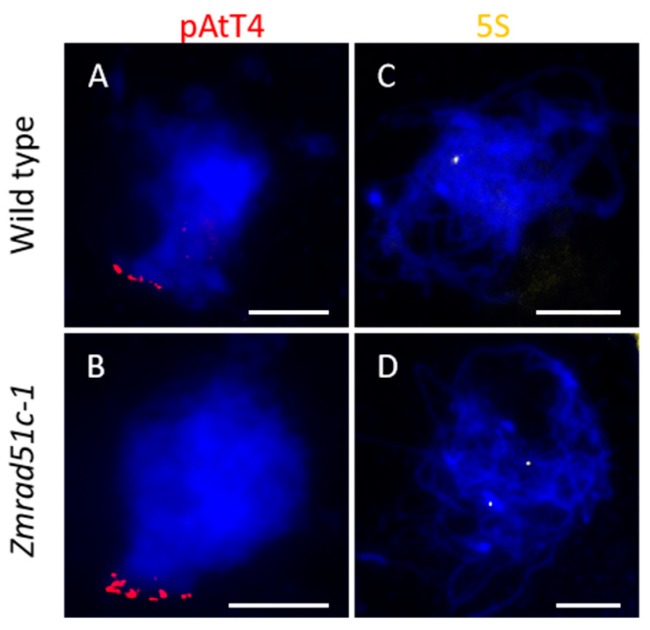
*ZmRAD51C* is not required for telomere bouquet clustering, but essential for homologous pairing. (**A,B**) FISH detection of telomere bouquet formation using pAtT4 probe in wild type (**A**) and *Zmrad51c-1* (**B**). DAPI staining was used to indicate the chromosomes. (**C,D**) FISH detection of homologous pairing using 5S rDNA probe in wild type (**C**) and *Zmrad51c-1* (**D**). Scale bars = 10 µm.

Telomere bouquet clustering is an evolutionarily conserved chromosome arrangement that clusters telomeres together to one side of the nucleus [56]. This specific structure may promote initiation of homologous pairing in early prophase I [57,58,59]. To explore whether mutation of *Zmrad51c* affects telomere bouquet formation, we conducted FISH (fluorescence in situ hybridization) analysis using pAtT4 as a telomere-specific probe in wild type and *Zmrad51c-1* meiocytes [60,61]. The results revealed that almost all of the pAtT4 signals were clustered together and attached to the nuclear envelope at early zygotene stage in both wild type (Figure 4A, *n* = 48) and *Zmrad51c-1* (Figure 4B, *n* = 55), indicating that *ZmRAD51C* is not required for telomere bouquet formation.

To explore whether the homologous chromosome pairing is defective in *Zmrad51c-1*, FISH analysis using 5S rDNA as a specific probe was conducted in both wild type and *Zmrad51c-1* meiocytes. The 5S ribosomal DNA (rDNA) is a tandem repetitive sequence located on the distal regions of the long arm of chromosome 2 and is often used to assess chromosome pairing and segregation [62,63]. In wild type, only one paired 5S rDNA signal was detected at pachytene (Figure 4C, *n* = 57), indicating that the homologous chromosomes were properly paired. In contrast, two separate 5S foci were consistently observed in nearly all of *Zmrad51c-1* meiocytes at this stage (Figure 4D, *n* = 60), indicating that homologous chromosome pairing was defective in *Zmrad51c-1* strain. Therefore, these results demonstrated that *ZmRAD51C* is essential for homologous chromosome pairing.

### 2.5. Normal Axial Element Installation but Defective Central Element Installation in Zmrad51c

**Figure 5:**
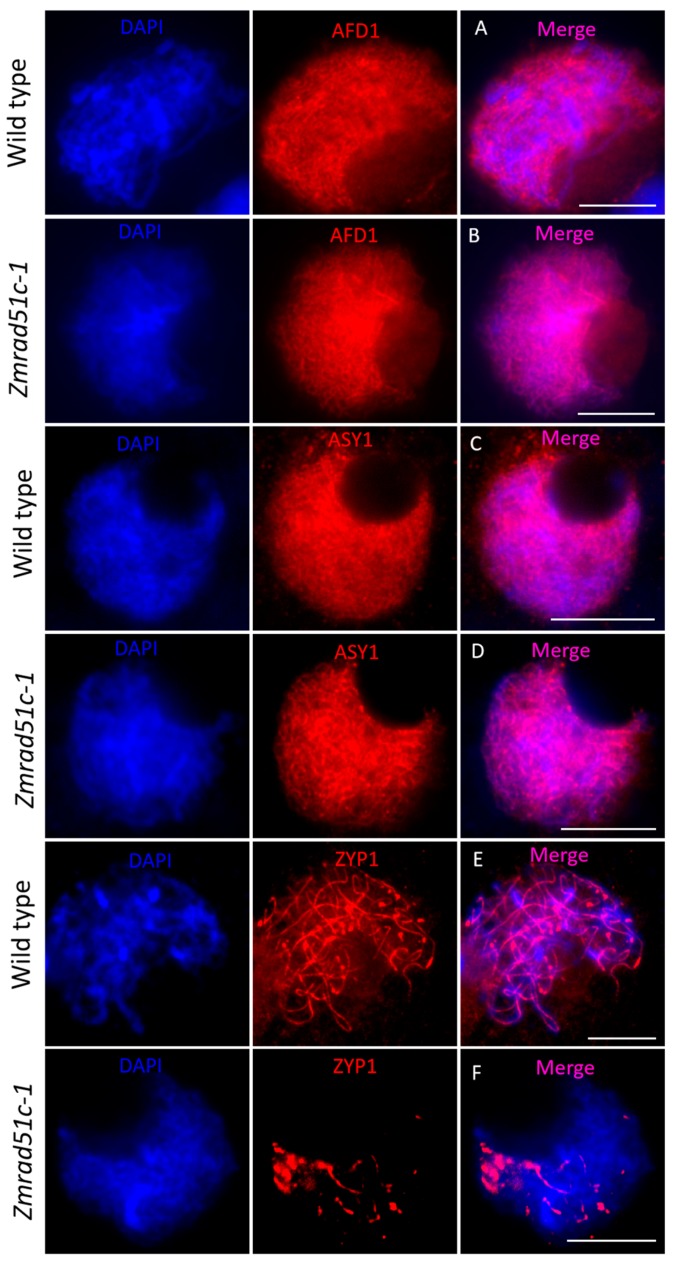
Immunolocalization of SC complex AFD1, ASY1, and ZYP1 proteins in wild type and *Zmrad51c-1*. Scale bars = 10 µm. DAPI staining was used to indicate the chromosomes. AFD1 (**A**), ASY1 (**C**), and ZYP1 (**E**) in wild type meiocytes; AFD1 (**B**), ASY1 (**D**), and ZYP1 (**F**) in *Zmrad51c-1* meiocytes.

The synaptonemal complex (SC) is a large protein scaffold connecting homologous chromosomes and is required for meiotic crossover formation to promote genetic information exchange between homologous chromosomes [64]. In order to monitor the installation behavior of the SC in *Zmrad51c-1*, we conducted immunolocalization using antibodies against AFD1, ASY1, and ZYP1 in wild type and *Zmrad51c-1*. AFD1, a critical component of the sister-chromatid cohesion complex (cohesion) associated with axial and lateral elements, is required for axial element (AE) elongation and maturation [65]. In wild type meiocytes, AFD1 behaved as linear signals along the entire chromosome from leptotene (Figure 5A, *n* = 11). In *Zmrad51c-1* meiocytes, the AFD1 loading pattern was consistent with wild type (Figure 5B, *n* = 14), indicating that *ZmRAD51C* is dispensable for AFD1 assembling. ASY1, an AE component of SC in maize, is the homolog of *S. cerevisiae* Hop1 and *Arabidopsis* ASY1, which is critical for homologous recombination and SC assembly [66,67]. In wild type, ASY1 loading appeared as long linear signals associated with chromosomes in zygotene (Figure 5C, *n* = 17). Similarly, the ASY1 signals distributed normally onto the chromosomes during zygotene in *Zmrad51c-1* mutant (Figure 5D, *n* = 13), indicating that *ZmRAD51C* is not required for AE installation. ZYP1, the central element (CE) component in maize, is a transverse filament (TF) protein that assembles between the AEs [67]. In wild type, ZYP1 signals were completely elongated along the entire length of synapsed chromosomes at pachytene (Figure 5E, *n* = 14). In contrast, only several scattered punctate or short stretches of ZYP1 signals were detected in *Zmrad51c-1* pachytene meiocytes (Figure 5F, *n* = 8). Quantification measurement and statistical analysis of ZYP1 length revealed that the ZYP1 length in *Zmrad51c-1* meiocytes was significantly shorter comparing with wild type (Figure S5). Taken together, these results indicate that *ZmRAD51C* is crucial for SC installation in maize meiosis.

### 2.6. Normal DSB Formation but Defective Loading of RAD51 and DMC1 in Zmrad51c

**Figure 6:**
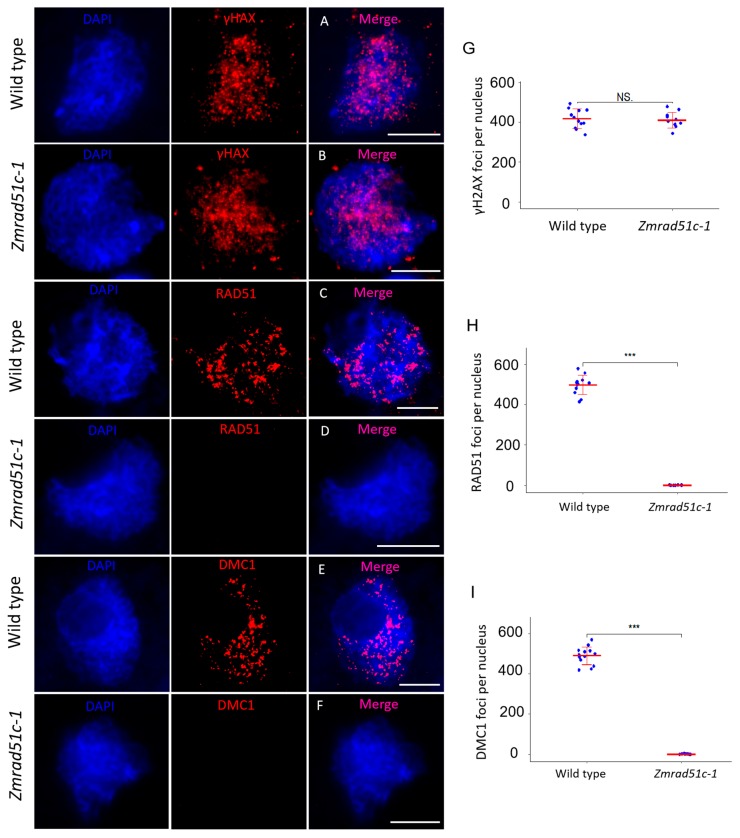
Immunolocalization of γH2AX, RAD51 and DMC1 antibodies on meiotic chromosomes in wild type and *Zmrad51c-1* meiocytes. (**A**,**B**) γH2AX foci in wild type (A) and *Zmrad51c-1* meiocytes (B). (**C**,**D**) RAD51 foci in wild type (C) and *Zmrad51c-1* meiocytes (D). (**E**,**F**) DMC1 foci in wild type (E) and *Zmrad51c-1* meiocytes (F). (**G**–**I**) Quantification and statistical analyses of γH2AX, RAD51, and DMC1 foci between wild type and *Zmrad51c-1* meiocytes. Triple asterisks indicate the statistical significant at *p* < 0.001 using a two-tailed Student’s *t*-test. NS means no significant difference.

In meiosis prophase I, the formation and repair of programmed DSBs is essential for HR [1]. Soon after DSBs are formed, a protein complex initiates histone H2AX phosphorylation (γH2AX) at the damaged site to promote DNA repair [68,69]. Therefore, γH2AX is often used as a biomarker to detect the appearance of DSBs [70]. To verify whether DSBs are formed normally in *Zmrad51c-1*, an antibody specifically recognizing plant γH2AX was raised in rabbit and used for immunolocalization in wild type and *Zmrad51c-1* meiocytes [71]. At zygotene, a substantial number of dot-like γH2AX signals was detected in both wild type (Figure 6A) and *Zmrad51c-1* meiocytes (Figure 6B), and the number of γH2AX foci in *Zmrad51c-1* (*n* = 12) were not significantly different from wild type (*n* = 11) (Figure 6G), suggesting that *ZmRAD51C* is dispensable for DSB formation.

To test whether *ZmRAD51C* functions in DSB repair, immunolocalization using antibodies against RAD51 and DMC1 proteins was conducted in wild type and *Zmrad51c-1* meiocytes. The loading of RAD51 and DMC1 onto the ssDNA is considered as an important cytological marker of HR-mediated DSB repair in different species [72,73]. In wild type, both RAD51 (Figure 6C and 6H, *n* = 12) and DMC1 (Figure 6E and 6I, *n* = 13) proteins were appeared as punctuate foci distributed on meiotic chromosomes in zygotene meiocytes. In contrast, no visible RAD51 (Figure 6D, *n* = 10) and DMC1 (Figure 6F, *n* = 12) foci were detected in *Zmrad51c-1* meiocytes at the same stage, indicating that *ZmRAD51C* is essential for meiotic DSB repair in maize. 

### 2.7. Zmrad51c Mutants Are Hypersensitive to DNA Damage Agent 

**Figure 7:**
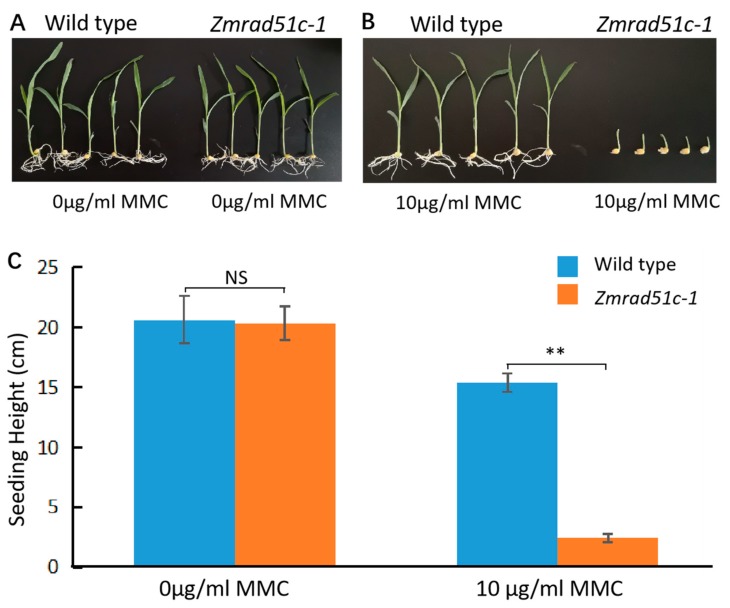
*Zmrad51-1* is hypersensitive to MMC. (**A**) Wild type and *Zmrad51c-1* showed similar height when growing on 1/2 MS medium without MMC for two weeks. (**B**) *Zmrad51-1* seedlings (right) were significantly shorter than wild type (left) on 1/2 MS medium treated with 10 μg/mL MMC for two weeks. (**C**) Statistical analysis of wild type (*n* = 10) and *Zmrad51-1* (*n* = 10) plant height. Values are means ± SE. Double asterisks indicates the statistical significance at *p* < 0.01 using a two-tailed Student’s *t*-test. NS means no significant difference.

Previous studies have shown that the DNA damage induced by the mitomycin C (MMC) was exclusively repaired by HR [74,75]. To investigate whether the mutation of *ZmRAD51*C could result in the defect in DSB repair during mitosis, the MMC was used to treat wild type and *Zmrad51c-1* from germination to seedlings. After genotyping and measuring the length of seedlings, we found that the plant height of *Zmrad51c-1* was significantly shorter than wild type when growing on 1/2 MS medium containing 10 μg/mL MMC (Figure 7), suggesting that *ZmRAD51C* may also play an important role in HR repair during mitosis.

## 3. Discussions

### 3.1. Functional Conservation of RAD51C in Different Organisms

Prior to this study, none of the RAD51 paralogues have been functionally characterized in maize yet. However, based on the high sequence identity with *Arabidopsis* and rice paralogs, we were be able to ascertain that there is a single copy gene encoding each of these five RAD51 paralogs in maize, respectively (Table S3). Meanwhile, the phylogeny analysis unambiguously discriminates that each of RAD51 paralogs fall into distinct clades (Figure S6), indicating that different RAD51 paralogues have divergent evolutionary origins. 

As one of the RAD51 paralogs, *RAD51C* has been known as an important player in DSB repair via HR in several organisms. In mammalian cells, deficiency in *RAD51C* results in spontaneous chromosome aberrations, hypersensitivity to DNA damaging agents, and early embryonic lethality [76,77,78,79,80]. In contrast, the loss-of-function of *RAD51C* in plants led to sterility without disturbing the vegetative growth under normal condition. Previous studies in *Arabidopsis* and rice demonstrated that both *AtRAD51C* and *OsRAD51C* are critically required for DSB repair in a manner downstream of DSB formation [31,40,45,46]. According to our study, the mutation in the maize *RAD51C* caused the complete sterility in both male and female meiosis, accompanied with an array of abnormal meiotic chromosome behaviors, including the lack of homologous pairing and synapsis, chromosome entanglement and fragmentation, and SC assembly failure. These results demonstrated that the *RAD51C* is also essential for meiotic DSB repair in maize. In addition, as no any RAD51 or DMC1 signals could be observed once *ZmRAD51C* disrupted, suggesting that either RAD51C functions by stabilizing the RAD51/DMC1-ssDNA nucleofilaments, or alternatively, RAD51C may operate directly upstream of RAD51 and DMC1 proteins to direct their loading onto chromosomes. Therefore, our findings strengthen the notion that the function of *RAD51C* in DSB repair is highly conserved among different species from mammal to plant. 

### 3.2. Abnormal Chromosome Fragmentation in Zmrad51c

Chromosome fragmentation is a characteristic phenomenon observed in mutants defective in DSB repair machinery. Our results showed that the *Zmrad51c* mutant phenotype is similar to that of the *Osrad51c* and *Atrad51c* mutants, as well as other related mutants such as *Osxrcc3* [44], *Osrad1* [81], *Osrad17* [82], *Atrad50* [83], and *Atmre11* [84]. However, the severity of the chromosome segregation seems to be much less in *Zmrad51c* compared to the *Osrad51c* and *Atrad51c* mutants. A possible explanation for this discrepancy could be that more or less in maize, the other RAD51 paralogues may function redundant with *ZmRAD51C*. Meanwhile, the other possibility we cannot exclude is that the other DSB repair pathway, such as non-homologous end-joining (NHEJ) [85] or microhomology-mediated end-joining (MMEJ) [86], may be alternatively motivated in the absence of the HR pathway at a different extent in maize relative to *Arabidopsis* and rice. In this context, it would be worthy to examine the meiotic alterations after combining the mutation in *ZmRAD51C* with mutations in genes involved in the NHEJ and MMEJ pathway, which have not been explored yet in maize.

## 4. Materials and Methods

### 4.1. Plant Materials

The *Zmrad51c-1* mutant was created by ethylmethane sulfonate (EMS) mutagenesis in the maize inbred line Mo17. A *Zmrad51c-2* mutant was obtained from the Maize EMS induced Mutant Database (MEMD, www.elabcaas.cn/memd/) [55]. Plants were cultivated and fertilized in a same manner as conventional maize production field during the growing season. 

### 4.2. Map-Based Cloning of Zmrad51c-1

To isolate the *Zmrad51c-1* gene, a positional cloning method was conducted. The heterozygote (*ZmRAD51C^+^/Zmrad51c^-^*) in inbred line Mo17 was outcrossed with maize inbred line B73 to generate F_1_, and then self-crossed to create a F_2_ segregation population. Completely infertility plants segregated from the F_2_ population were used for fine mapping. InDel (insertion-deletion) and SNP markers were designed based on the sequence divergence between the B73 and Mo17 inbred lines [87,88]. Primer sequences used in cloning and mutants genotyping are listed in Table S1.

### 4.3. cDNA Cloning and Real-Time qPCR for Transcript Expression Assay

Total RNA was extracted individually from root, stem, leaf, developing embryo, and endosperm as well as young tassel and young ear. Reverse transcription was conducted using PrimeScriptTM Ⅱ 1st strand cDNA Synthesis Kit (TaKaRa, Tokyo, Japan) with Oligo-T as primer. The full-length CDS was amplified by the TransStart FastPfu Fly DNA Polymerase kit (TransGen) using 1st strand cDNA as a template. Quantitative real-time PCR analyses were conducted through a 7500 Fast Real-Time PCR System (Applied Biosystem, Foster city, CA, USA) using SYBR green detection protocol (TaKaRa). Gene-specific primers and ubiquitin gene primers for the control standard were used in the RT-qPCR. The primer sequences used in cDNA synthesis and RT-qPCR are listed in Table S2.

### 4.4. Pollen Viability Observation

Pollen viability was detected by Alexander staining, following previous protocols [54,89]. Mature anthers were dissected from the wild type and *Zmrad51c* mutants at tasseling stage. Pollen grains were separated from anthers using forceps and blades, then stained with 10% Alexander solution and observed in a Leica EZ4 HD stereo-microscope. The pictures of pollen grains were taken under a Leica DM2000 LED (Leica, Solms, Germany)

### 4.5. Meiotic Chromosome Preparation

Immature tassels of wild type and mutants were harvested and fixed in Carnoy’s solution (ethanol:glacial acetic; 3:1) and stored in 70% ethanol at 4 °C. Anthers at proper stages were dissected and squashed in a 45% acetocarmine solution. Slides with chromosomes were frozen in liquid nitrogen, then the coverslip was removed rapidly. Slides were dehydrated in different ethanol concentration (70%, 90%, and 100%) and air-dried. DAPI in an anti-fade solution (Vector laboratories, Burlingame, CA, USA) was used to counterstain chromosomes. Images were captured using a DS-Qi2 Microscope Camera system installed in a Ci-S-FL microscope (Nikon, Tokyo, Japan).

### 4.6. FISH Analysis

FISH analysis was performed, as described previously [48]. The pTa794 clone containing 5S rDNA repeats or pAtT4 clone containing telomeric-specific repeats were used as FISH probes [60,90]. Probes were labeled with fluorophore-dUTP (Roche, Basel, Switzerland) by nick translation. Anti-digoxigenin (Vector Laboratories) was used as the second antibody for detection with a probe of digoxigenin (DIG). Chromosomes were counterstained using Antifade Mounting Medium with DAPI (Vector laboratories). Finally, chromosome images were taken using a DS-Qi2 Microscope Camera system installed in a Ci-S-FL microscope (Nikon).

### 4.7. Fluorescence Immunolocalization Assays

Immunolocalization assays were conducted, as previous described [72]. Fresh young anthers were dissected from immature tassel and fixed in 1× Buffer A containing 4% (*w*/*v*) paraformaldehyde for 30 min at room temperature (25 °C), then shake washed in 1× Buffer A at room temperature for twice and stored in 1× Buffer A at 4 °C. Anthers at proper stages were dissected in 1× Buffer A solution to release meiocytes, then squashed and frozen in liquid nitrogen to rapidly remove the coverslip. Subsequently, the slides were incubated with different antibodies diluted in blocking buffer in a humidity chamber at 37 °C for 1 h and washed 3 times in 1× PBS. Alexa Fluor 555-conjugated goat anti-rabbit antibodies were added to the slides. After washed for 3 times in 1× PBS, the slides were counterstained with DAPI in antifade solution. The slides were observed and images were taken by a Ci-S-FL microscope (Nikon) to generate 2D projected images. Surface rendered images were colored and merged by the ImageJ software (https://imagej.nih.gov/ij/index.html).

The polyclonal antibodies against ASY1, ZYP1, and γH2AX were prepared by specific sequence fusion-peptide immunization in rabbit. AFD1, RAD51, and DMC1 antibodies were gifts from Changbin Chen (University of Minnesota, Twin Cities, MN, United State), Wojciech Pawlowski (Cornell University, Ithaca, NY, United State) and Huabang Chen (Institute of Genetics and Developmental Biology, Beijing, China), respectively. 

### 4.8. DNA Damage Agent Mitomycin C (MMC) Treatment

Self-pollinated seeds of heterozygous *ZmRAD51C-1^+/–^* plants were surface-sterilized in 75% ethanol for 10 min and 5% NaClO for 15 min, and soaked in sterilized water for one day. Then the sterilized seeds were sown on 1/2 MS solid medium containing 0 or 10 μg/mL MMC (Biochempartner, Wuhan, China) and grown in a growth chamber with a photoperiod cycle of 16 h light in 28 °C and 8 h darkness in 25 °C. Genotyping and phenotyping of the seedlings were conducted two weeks after the seeds were sown. 

### 4.9. Image Statistical Analysis

To quantitate the ZYP1 length and the γH2AX, RAD51 and DMC1 foci numbers in wild type and *Zmrad51c-1*, images were analyzed using ImageJ software based on the measuring instructions of NIH (https://imagej.nih.gov/ij/docs/pdfs/ImageJ.pdf). Statistical significance was calculated using two-tailed Student’s *t*-tests. Scatter Plots were drawn by R language using the “ggplot2” package. 

## Supplementary Materials

Supplementary materials can be found at https://www.mdpi.com/1422-0067/20/21/5513/s1.

## Abbreviations

DSB	Double strand break
HR	Homologous recombination
SC	Synaptonemal complex
AE	Axial elements
CE	Central elements
TF	Transverse filament

## References

[1] De Massy B. (2013). Initiation of meiotic recombination: How and where? Conservation and specificities among eukaryotes. Annu. Rev. Genet..

[2] Edlinger B., Schlogelhofer P. (2011). Have a break: Determinants of meiotic DNA double strand break (DSB) formation and processing in plants. J. Exp. Bot..

[3] Keeney S. (2008). Spo11 and the Formation of DNA Double-Strand Breaks in Meiosis. Genome Dyn. Stab..

[4] Keeney S., Neale M.J. (2006). Initiation of meiotic recombination by formation of DNA double-strand breaks: Mechanism and regulation. Biochem Soc. Trans..

[5] Cole F., Keeney S., Jasin M. (2010). Evolutionary conservation of meiotic DSB proteins: More than just Spo11. Genes Dev..

[6] Alani E., Padmore R., Kleckner N. (1990). Analysis of wild-type and rad50 mutants of yeast suggests an intimate relationship between meiotic chromosome synapsis and recombination. Cell.

[7] Cao L., Alani E., Kleckner N. (1990). A pathway for generation and processing of double-strand breaks during meiotic recombination in *S. cerevisiae*. Cell.

[8] Ivanov E.L., Korolev V.G., Fabre F. (1992). XRS2, a DNA repair gene of Saccharomyces cerevisiae, is needed for meiotic recombination. Genetics.

[9] McKee A.H., Kleckner N. (1997). A general method for identifying recessive diploid-specific mutations in Saccharomyces cerevisiae, its application to the isolation of mutants blocked at intermediate stages of meiotic prophase and characterization of a new gene SAE2. Genetics.

[10] Nairz K., Klein F. (1997). mre11S—A yeast mutation that blocks double-strand-break processing and permits nonhomologous synapsis in meiosis. Genes Dev..

[11] Prinz S., Amon A., Klein F. (1997). Isolation of COM1, a new gene required to complete meiotic double-strand break-induced recombination in Saccharomyces cerevisiae. Genetics.

[12] Longhese M.P., Bonetti D., Guerini I., Manfrini N., Clerici M. (2009). DNA double-strand breaks in meiosis: Checking their formation, processing and repair. DNA Repair.

[13] Mimitou E.P., Symington L.S. (2009). DNA end resection: Many nucleases make light work. DNA Repair.

[14] Bishop D.K., Park D., Xu L., Kleckner N. (1992). DMC1: A meiosis-specific yeast homolog of *E. coli* recA required for recombination, synaptonemal complex formation, and cell cycle progression. Cell.

[15] Shinohara A., Ogawa H., Ogawa T. (1992). Rad51 protein involved in repair and recombination in *S. cerevisiae* is a RecA-like protein. Cell.

[16] Cloud V., Chan Y.L., Grubb J., Budke B., Bishop D.K. (2012). Rad51 is an accessory factor for Dmc1-mediated joint molecule formation during meiosis. Science.

[17] Allers T., Lichten M. (2001). Differential timing and control of noncrossover and crossover recombination during meiosis. Cell.

[18] Borner G.V., Kleckner N., Hunter N. (2004). Crossover/noncrossover differentiation, synaptonemal complex formation, and regulatory surveillance at the leptotene/zygotene transition of meiosis. Cell.

[19] Seitz E.M., Brockman J.P., Sandler S.J., Clark A.J., Kowalczykowski S.C. (1998). RadA protein is an archaeal RecA protein homolog that catalyzes DNA strand exchange. Genes Dev..

[20] Baumann P., West S.C. (1998). Role of the human RAD51 protein in homologous recombination and double-stranded-break repair. Trends Biochem. Sci..

[21] Shinohara A., Ogawa H., Matsuda Y., Ushio N., Ikeo K., Ogawa T. (1993). Cloning of human, mouse and fission yeast recombination genes homologous to RAD51 and recA. Nat. Genet..

[22] Tebbs R.S., Zhao Y., Tucker J.D., Scheerer J.B., Siciliano M.J., Hwang M., Liu N., Legerski R.J., Thompson L.H. (1995). Correction of chromosomal instability and sensitivity to diverse mutagens by a cloned cDNA of the XRCC3 DNA repair gene. Proc. Natl. Acad. Sci. USA.

[23] Albala J.S., Thelen M.P., Prange C., Fan W., Christensen M., Thompson L.H., Lennon G.G. (1997). Identification of a novel human RAD51 homolog, RAD51B. Genomics.

[24] Dosanjh M.K., Collins D.W., Fan W., Lennon G.G., Albala J.S., Shen Z., Schild D. (1998). Isolation and characterization of RAD51C, a new human member of the RAD51 family of related genes. Nucl. Acids Res..

[25] Liu N., Lamerdin J.E., Tebbs R.S., Schild D., Tucker J.D., Shen M.R., Brookman K.W., Siciliano M.J., Walter C.A., Fan W. (1998). XRCC2 and XRCC3, new human Rad51-family members, promote chromosome stability and protect against DNA cross-links and other damages. Mol. Cell.

[26] Pittman D.L., Weinberg L.R., Schimenti J.C. (1998). Identification, characterization, and genetic mapping of Rad51d, a new mouse and human RAD51/RecA-related gene. Genomics.

[27] Schild D., Lio Y.C., Collins D.W., Tsomondo T., Chen D.J. (2000). Evidence for simultaneous protein interactions between human Rad51 paralogs. J. Biol. Chem..

[28] Masson J.Y., Tarsounas M.C., Stasiak A.Z., Stasiak A., Shah R., McIlwraith M.J., Benson F.E., West S.C. (2001). Identification and purification of two distinct complexes containing the five RAD51 paralogs. Genes Dev..

[29] Liu N., Schild D., Thelen M.P., Thompson L.H. (2002). Involvement of Rad51C in two distinct protein complexes of Rad51 paralogs in human cells. Nucl. Acids Res..

[30] Miller K.A., Sawicka D., Barsky D., Albala J.S. (2004). Domain mapping of the Rad51 paralog protein complexes. Nucl. Acids Res..

[31] Li W., Yang X., Lin Z., Timofejeva L., Xiao R., Makaroff C.A., Ma H. (2005). The AtRAD51C gene is required for normal meiotic chromosome synapsis and double-stranded break repair in Arabidopsis. Plant Physiol..

[32] Liu Y., Masson J.Y., Shah R., O’Regan P., West S.C. (2004). RAD51C is required for Holliday junction processing in mammalian cells. Science.

[33] Kuznetsov S., Pellegrini M., Shuda K., Fernandez-Capetillo O., Liu Y., Martin B.K., Burkett S., Southon E., Pati D., Tessarollo L. (2007). RAD51C deficiency in mice results in early prophase I arrest in males and sister chromatid separation at metaphase II in females. J. Cell Biol..

[34] Suwaki N., Klare K., Tarsounas M. (2011). RAD51 paralogs: Roles in DNA damage signalling, recombinational repair and tumorigenesis. Semin. Cell Dev. Biol..

[35] Lio Y.C., Mazin A.V., Kowalczykowski S.C., Chen D.J. (2003). Complex formation by the human Rad51B and Rad51C DNA repair proteins and their activities in vitro. J. Biol. Chem..

[36] Wiese C., Collins D.W., Albala J.S., Thompson L.H., Kronenberg A., Schild D. (2002). Interactions involving the Rad51 paralogs Rad51C and XRCC3 in human cells. Nucl. Acids Res..

[37] Masson J.Y., Stasiak A.Z., Stasiak A., Benson F.E., West S.C. (2001). Complex formation by the human RAD51C and XRCC3 recombination repair proteins. Proc. Natl. Acad. Sci. USA.

[38] Bishop D.K., Ear U., Bhattacharyya A., Calderone C., Beckett M., Weichselbaum R.R., Shinohara A. (1998). Xrcc3 is required for assembly of Rad51 complexes in vivo. J. Biol. Chem..

[39] Lin Z., Kong H., Nei M., Ma H. (2006). Origins and evolution of the recA/RAD51 gene family: Evidence for ancient gene duplication and endosymbiotic gene transfer. Proc. Natl. Acad. Sci. USA.

[40] Abe K., Osakabe K., Nakayama S., Endo M., Tagiri A., Todoriki S., Ichikawa H., Toki S. (2005). Arabidopsis RAD51C gene is important for homologous recombination in meiosis and mitosis. Plant Physiol..

[41] Osakabe K., Yoshioka T., Ichikawa H., Toki S. (2002). Molecular cloning and characterization of RAD51-like genes from Arabidopsis thaliana. Plant Mol. Biol..

[42] Bleuyard J.Y., Gallego M.E., Savigny F., White C.I. (2005). Differing requirements for the Arabidopsis Rad51 paralogs in meiosis and DNA repair. Plant J..

[43] Bleuyard J.Y., White C.I. (2004). The Arabidopsis homologue of Xrcc3 plays an essential role in meiosis. EMBO J..

[44] Zhang B., Wang M., Tang D., Li Y., Xu M., Gu M., Cheng Z., Yu H. (2015). XRCC3 is essential for proper double-strand break repair and homologous recombination in rice meiosis. J. Exp. Bot..

[45] Tang D., Miao C., Li Y., Wang H., Liu X., Yu H., Cheng Z. (2014). OsRAD51C is essential for double-strand break repair in rice meiosis. Front. Plant. Sci..

[46] Kou Y., Chang Y., Li X., Xiao J., Wang S. (2012). The rice RAD51C gene is required for the meiosis of both female and male gametocytes and the DNA repair of somatic cells. J. Exp. Bot..

[47] Strable J., Scanlon M.J. (2009). Maize (Zea mays): A model organism for basic and applied research in plant biology. Cold Spring Harb. Protoc..

[48] Wang C.J., Harper L., Cande W.Z. (2006). High-resolution single-copy gene fluorescence in situ hybridization and its use in the construction of a cytogenetic map of maize chromosome 9. Plant Cell.

[49] Pawlowski W.P. (2010). Chromosome organization and dynamics in plants. Curr. Opin. Plant Biol..

[50] Wang C.J. (2013). Analyzing maize meiotic chromosomes with super-resolution structured illumination microscopy. Methods Mol. Biol..

[51] Chang P., Tseng Y.F., Chen P.Y., Wang C.R. (2018). Using Flow Cytometry to Isolate Maize Meiocytes for Next Generation Sequencing: A Time and Labor Efficient Method. Curr. Protoc. Plant. Biol..

[52] Chang M.T., Neuffer M.G., Freeling M., Walbot V. (1994). Chromosomal Behavior During Microsporogenesis. The Maize Handbook.

[53] Timofejeva L., Skibbe D.S., Lee S., Golubovskaya I., Wang R., Harper L., Walbot V., Cande W.Z. (2013). Cytological characterization and allelism testing of anther developmental mutants identified in a screen of maize male sterile lines. G3 (BethesdaMd.).

[54] Alexander M.P. (1969). Differential staining of aborted and nonaborted pollen. Stain Technol..

[55] Lu X., Liu J., Ren W., Yang Q., Chai Z., Chen R., Wang L., Zhao J., Lang Z., Wang H. (2018). Gene-Indexed Mutations in Maize. Mol. Plant.

[56] Blokhina Y.P., Nguyen A.D. (2019). The telomere bouquet is a hub where meiotic double-strand breaks, synapsis, and stable homolog juxtaposition are coordinated in the zebrafish, Danio rerio. PLoS Genet..

[57] Golubovskaya I.N., Harper L.C., Pawlowski W.P., Schichnes D., Cande W.Z. (2002). The pam1 gene is required for meiotic bouquet formation and efficient homologous synapsis in maize (*Zea mays* L.). Genetics.

[58] Harper L., Golubovskaya I., Cande W.Z. (2004). A bouquet of chromosomes. J. Cell Sci..

[59] Klutstein M., Fennell A., Fernandez-Alvarez A., Cooper J.P. (2015). The telomere bouquet regulates meiotic centromere assembly. Nat. Cell Biol..

[60] Richards E.J., Ausubel F.M. (1988). Isolation of a higher eukaryotic telomere from Arabidopsis thaliana. Cell.

[61] Prieto P., Martin A., Cabrera A. (2004). Chromosomal distribution of telomeric and telomeric-associated sequences in Hordeum chilense by in situ hybridization. Hereditas.

[62] Li L., Arumuganathan K. (2001). Physical mapping of 45S and 5S rDNA on maize metaphase and sorted chromosomes by FISH. Hereditas.

[63] Kato A., Lamb J.C., Birchler J.A. (2004). Chromosome painting using repetitive DNA sequences as probes for somatic chromosome identification in maize. Proc. Natl. Acad. Sci. USA.

[64] Cahoon C.K., Hawley R.S. (2016). Regulating the construction and demolition of the synaptonemal complex. Nat. Struct. Mol. Biol..

[65] Golubovskaya I.N., Hamant O., Timofejeva L., Wang C.J., Braun D., Meeley R., Cande W.Z. (2006). Alleles of afd1 dissect REC8 functions during meiotic prophase I. J. Cell Sci..

[66] Armstrong S.J., Caryl A.P., Jones G.H., Franklin F.C. (2002). Asy1, a protein required for meiotic chromosome synapsis, localizes to axis-associated chromatin in Arabidopsis and Brassica. J. Cell Sci..

[67] Golubovskaya I.N., Wang C.J., Timofejeva L., Cande W.Z. (2011). Maize meiotic mutants with improper or non-homologous synapsis due to problems in pairing or synaptonemal complex formation. J. Exp. Bot..

[68] Geric M., Gajski G., Garaj-Vrhovac V. (2014). gamma-H2AX as a biomarker for DNA double-strand breaks in ecotoxicology. Ecotoxicol. Environ. Saf..

[69] Turinetto V., Giachino C. (2015). Multiple facets of histone variant H2AX: A DNA double-strand-break marker with several biological functions. Nucl. Acids Res..

[70] Valdiglesias V., Giunta S., Fenech M., Neri M., Bonassi S. (2013). gammaH2AX as a marker of DNA double strand breaks and genomic instability in human population studies. Mutat. Res..

[71] Friesner J.D., Liu B., Culligan K., Britt A.B. (2005). Ionizing radiation-dependent gamma-H2AX focus formation requires ataxia telangiectasia mutated and ataxia telangiectasia mutated and Rad3-related. Mol. Biol. Cell.

[72] Pawlowski W.P., Golubovskaya I.N., Cande W.Z. (2003). Altered nuclear distribution of recombination protein RAD51 in maize mutants suggests the involvement of RAD51 in meiotic homology recognition. Plant. Cell.

[73] Etedali F., Baghban Kohnehrouz B., Valizadeh M., Gholizadeh A., Malboobi M.A. (2011). Genome wide cloning of maize meiotic recombinase Dmc1 and its functional structure through molecular phylogeny. Genet. Mol. Res. GMR.

[74] De Silva I.U., McHugh P.J., Clingen P.H., Hartley J.A. (2002). Defects in interstrand cross-link uncoupling do not account for the extreme sensitivity of ERCC1 and XPF cells to cisplatin. Nucl. Acids Res..

[75] Sasaki M.S., Takata M., Sonoda E., Tachibana A., Takeda S. (2004). Recombination repair pathway in the maintenance of chromosomal integrity against DNA interstrand crosslinks. Cytogenet. Genome Res..

[76] Drexler G.A., Rogge S., Beisker W., Eckardt-Schupp F., Zdzienicka M.Z., Fritz E. (2004). Spontaneous homologous recombination is decreased in Rad51C-deficient hamster cells. DNA Repair.

[77] Kuznetsov S.G., Haines D.C., Martin B.K., Sharan S.K. (2009). Loss of Rad51c leads to embryonic lethality and modulation of Trp53-dependent tumorigenesis in mice. Cancer Res..

[78] Smeenk G., de Groot A.J., Romeijn R.J., van Buul P.P., Zdzienicka M.Z., Mullenders L.H., Pastink A., Godthelp B.C. (2010). Rad51C is essential for embryonic development and haploinsufficiency causes increased DNA damage sensitivity and genomic instability. Mutat. Res..

[79] Min A., Im S.A., Yoon Y.K., Song S.H., Nam H.J., Hur H.S., Kim H.P., Lee K.H., Han S.W., Oh D.Y. (2013). RAD51C-deficient cancer cells are highly sensitive to the PARP inhibitor olaparib. Mol. Cancer Ther..

[80] Yonetani Y., Hochegger H., Sonoda E., Shinya S., Yoshikawa H., Takeda S., Yamazoe M. (2005). Differential and collaborative actions of Rad51 paralog proteins in cellular response to DNA damage. Nucl. Acids Res..

[81] Hu Q., Tang D., Wang H. (2016). The Exonuclease Homolog OsRAD1 Promotes Accurate Meiotic Double-Strand Break Repair by Suppressing Nonhomologous End Joining. Plant Physiol..

[82] Hu Q., Zhang C., Xue Z., Ma L., Liu W., Shen Y., Ma B., Cheng Z. (2018). OsRAD17 Is Required for Meiotic Double-Strand Break Repair and Plays a Redundant Role With OsZIP4 in Synaptonemal Complex Assembly. Front. Plant. Sci..

[83] Vannier J.B., Depeiges A., White C., Gallego M.E. (2006). Two roles for Rad50 in telomere maintenance. EMBO J..

[84] Samanic I., Simunic J., Riha K., Puizina J. (2013). Evidence for distinct functions of MRE11 in Arabidopsis meiosis. PLoS ONE.

[85] McVey M., Lee S.E. (2008). MMEJ repair of double-strand breaks (director’s cut): Deleted sequences and alternative endings. Trends Genet..

[86] Shrivastav M., De Haro L.P., Nickoloff J.A. (2008). Regulation of DNA double-strand break repair pathway choice. Cell Res..

[87] Yang N., Xu X.W., Wang R.R., Peng W.L., Cai L., Song J.M., Li W., Luo X., Niu L., Wang Y. (2017). Contributions of Zea mays subspecies mexicana haplotypes to modern maize. Nat. Commun..

[88] Sun S., Zhou Y., Chen J., Shi J. (2018). Extensive intraspecific gene order and gene structural variations between Mo17 and other maize genomes. Nat. Genet..

[89] Johnson-Brousseau S.A., McCormick S. (2004). A compendium of methods useful for characterizing Arabidopsis pollen mutants and gametophytically-expressed genes. Plant. J..

[90] Ji J., Tang D., Wang K., Wang M., Che L., Li M., Cheng Z. (2012). The role of OsCOM1 in homologous chromosome synapsis and recombination in rice meiosis. Plant. J..

