# Supplementary material for: ZmRAD51C Is Essential for Double-Strand Break Repair and Homologous Recombination in Maize Meiosis
Source: Int J Mol Sci. 2019 Nov 5;20(21):5513. doi: 10.3390/ijms20215513 (PMC6861927; doi:10.3390/ijms20215513)
Supplement: Supplementary file 1 [file ijms-20-05513-s001.zip › ijms-606547-revised-r2supplementary/Table S1. Primers designed for map-based cloning and mutants genotyping .doc]

**Table S1.** Primers designed for map-based cloning and mutants genotyping

| Name | Forward (5’-3’) | Reverse (5’-3’) |
| --- | --- | --- |
| **For Map-based cloning** | | |
| M152.3 | GATCAGGGCAGAGGCAACTAGGG | ATGAACACCACGCACTCAACTTTT |
| M177.4 | AAAGGACGAATGCGGCATCAAG | GGGGCAGGACGATTGGTAGTAAG |
| M198.9 | TCTCGGGATTTTTCTTGGTCTGG | TGGTTTATCGTGGAGAGAGAGGC |
| M205.6 | GGTATGGGCAAAGACTACATCAAAC | AGGACATGGTATCTTTGACCCTTAT |
| M217.4 | AGAAATGGACCATTGTAAGGCTATG | CTTTCCGCTTAAGTGTTGCTTTA |
| M219.40 | GGCCAGGTGGTAGATAAGAACATA | GCCAGCTTGACAGGGATAGTTTT |
| M219.69 | GACAACAGGAACACTCAGATTTGGT | ACACAGGATTTGGATAAGTTTAGCC |
| M220.17 | ACTGGGAGTCAAGTGGAA | AGTGGTGGGATAGTGGTT |
| M220.96 | TAATCTGGGCGCAAACGCAGGAACT | GCCGACGCCAAGACATACCCTA |
| M221.5 | AGATCGATGTGCATTCCAAAAGA | CCAAAAAACGGTAGCTTTTGATC |
| M230.5 | AGTACAAGCAGAGTGCATCTTTAGA | TTTTGTTGCATAAACCTCGCTGGT |
| **For mutants genotyping** | | |
| RAD51C1-seq | CTGGTGACCTCAATGACAT | CAGAAGAGGACTTCTCATGG |
| RAD51C2-seq | CAAATCCCAGTGGAATGTG | CAGAAGAGGACTTCTCATGG |
| RAD51C2-RT | ACTGCCTGATGCACAGCACT | GCTCTCTGAGGAACTTCTCC |
