# Supplementary material for: ZmRAD51C Is Essential for Double-Strand Break Repair and Homologous Recombination in Maize Meiosis
Source: Int J Mol Sci. 2019 Nov 5;20(21):5513. doi: 10.3390/ijms20215513 (PMC6861927; doi:10.3390/ijms20215513)
Supplement: Supplementary file 1 [file ijms-20-05513-s001.zip › ijms-606547-revised-r2supplementary/Table S3 List of the putative RAD51 paralogues in maize..docx]

**Table S3 List of the putative RAD51 paralogues in maize.**

| **Gene** | **Gene ID** | **Identity of protein sequence with rice homolog** | **Identity of protein sequence with Arabidopsis homolog** |
| --- | --- | --- | --- |
| RAD51B | Zm00001d010986 | 76% | 55% |
| RAD51C | Zm00001d044278 | 83% | 66% |
| RAD51D | Zm00001d022332 | 67% | 49% |
| XRCC2 | Zm00001d042691 | 67% | 39% |
| XRCC3 | Zm00001d016839 | 83% | 43% |
