# Supplementary material for: ZmRAD51C Is Essential for Double-Strand Break Repair and Homologous Recombination in Maize Meiosis
Source: Int J Mol Sci. 2019 Nov 5;20(21):5513. doi: 10.3390/ijms20215513 (PMC6861927; doi:10.3390/ijms20215513)
Supplement: Supplementary file 1 [file ijms-20-05513-s001.zip › ijms-606547-revised-r2supplementary/Table S2. Primers designed for full-length cDNA amplification and qRT-PCR.doc]

**Table S2.** Primers designed for full-length cDNA amplification and qRT-PCR

| Name | Forward (5’-3’) | Reverse (5’-3’) |
| --- | --- | --- |
| **For full-length cDNA amplification** | | |
| RAD51C-flc | TTCTCTGATGAGCTGTCAC | CTACGTTACTCGGGCTCGCTT |
| **For qRT-PCR** | | |
| RAD51C-RT | TTCTCTGATGAGCTGTCAC | CCAGTTGAGTTTTACCAACC |
| ZmUBQ | TAAGCTGCCGATGTGCCTGCG | CTGAAAGACAGAACATAATGAGCACA |
